# A Hardware–Software Integrated PCB Image Registration Method Based on Local Adaptive KNN and SIFT

**DOI:** 10.3390/s26154858

**Published:** 2026-08-01

**Authors:** Wenjie Su, En Fan, Jilong Wang, Siyu Ling, Zhaoxi Fang

**Affiliations:** 1 Institute of Artificial Intelligence, Shaoxing University, Shaoxing 312000, China; 23136123@usx.edu.cn (W.S.); fangzhaoxi@usx.edu.cn (Z.F.); 2 Zhejiang Ningwei Machinery Technology Co., Ltd., Shaoxing 311814, China; wjl@zjjilong.com; 3 School of Physics, Shaoxing University, Shaoxing 312000, China; 23517210@usx.edu.cn

**Keywords:** PCB, image registration, SIFT, K-nearest neighbors, adaptive threshold, hardware–software integration

## Abstract

Solder-joint detection and localization on large, complex printed circuit boards (PCBs) remain challenging because PCB images often contain unevenly distributed features, nonuniform illumination, specular reflection, scale variation and geometric distortion. Conventional SIFT-based registration methods usually use fixed matching parameters and therefore cannot adapt well to regions with different component densities. To address this limitation, this study proposes a hardware–software integrated PCB image registration framework that combines a robotic end effector with a locally adaptive K-nearest neighbor (LAKNN) strategy and SIFT descriptors. The hardware platform provides stable image acquisition through a lifting mechanism and ring-light illumination, while the software module adjusts the neighbor-search space according to local feature density and matching confidence. Experimental results show that the proposed LAKNN method achieves 90.8% inlier-match accuracy in the ablation experiment and improves registration robustness under height, region and viewpoint variations. The proposed framework provides a practical basis for automated PCB inspection and robotic soldering alignment.

## 1. Introduction

With the rapid development of intelligent manufacturing, the production and inspection of electronic products are becoming increasingly automated. As a core component of electronic products, the printed circuit board (PCB) directly affects product reliability, assembly quality and downstream inspection efficiency [1]. Accurate solder-joint localization and PCB image registration are therefore essential for automated soldering and quality control. However, large PCB images usually contain repeated pads, similar textures, uneven feature distributions and scale or viewpoint changes, which make robust registration difficult [2,3].

In PCB inspection, image registration is commonly used to align a reference image, such as a design drawing or standard template, with a sensed image captured during production [4,5,6]. Existing approaches include template matching, multi-view stereo methods and laser-scanning-based methods [7]. Multi-template and pyramid-based methods can improve local matching of soldering paths [8], while feature-descriptor methods, such as SIFT and ORB, combined with RANSAC can improve robustness under rotation and scale changes [9]. Nevertheless, these approaches still suffer from parameter sensitivity and mismatches when a large PCB contains repeated local patterns.

Deep learning methods, including convolutional neural networks (CNNs) [10] and object-detection frameworks such as Faster R-CNN [11], have achieved promising results in general detection and matching tasks. However, they are less suitable for the present industrial scenario for two reasons. First, robust training usually requires a large annotated dataset covering board types, solder-joint appearances, lighting conditions and defect variations. Second, inference on edge devices used in cost-sensitive production environments can impose considerable computational and deployment costs. In contrast, the proposed LAKNN–SIFT method is training-free, interpretable and lightweight, making it more suitable for flexible small-batch and high-mix PCB manufacturing.

Feature-point-based registration methods, especially SIFT and SURF [12,13], have been widely used because they extract local key points and descriptors that are relatively robust to scale and rotation. In a typical SIFT workflow, K-nearest neighbor (KNN) matching is used to find candidate correspondences [14], followed by outlier rejection using geometric constraints such as RANSAC [15]. However, conventional KNN uses a fixed K value or a fixed ratio threshold, which is not optimal for large PCB images. Dense pin regions, sparse substrate regions and repeated solder joints have very different local feature distributions, causing either false matches or missed matches when fixed parameters are used [16,17,18].

In practical large-PCB alignment, two issues are particularly important. First, the PCB may not be fixed at an identical position or viewing angle, resulting in rotation, perspective and scale variations. Second, solder joints and pads often have repeated textures and uneven spatial density, leading to ambiguous descriptor matches [19,20,21]. To address these problems, this paper proposes an image registration method that integrates SIFT feature extraction with a locally adaptive KNN matching strategy. The main contribution is to adapt the matching process according to local feature density and descriptor-level matching confidence, thereby improving registration accuracy in feature-rich and feature-sparse PCB regions.

The remainder of this paper is organized as follows. Section 2 describes the hardware experimental system and image-processing workflow. Section 3 presents the traditional baseline methods and the proposed LAKNN–SIFT registration method. Section 4 reports the experimental setup, results and ablation analysis. Section 5 concludes the paper and outlines future work.

## 2. System Architecture and Main Functions

**Figure 1:**
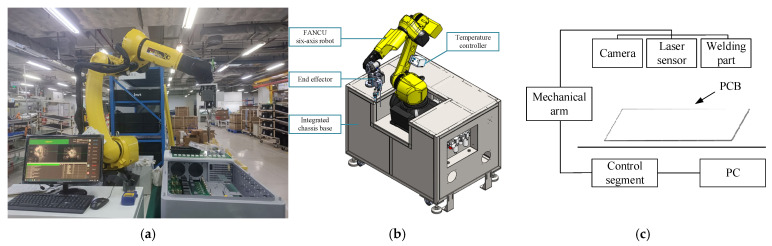
Large-scale PCB robotic welding system; (**a**) physical drawing of the automatic soldering robot; (**b**) schematic diagram of the soldering machine structure; (**c**) soldering system architecture.

**Figure 2:**
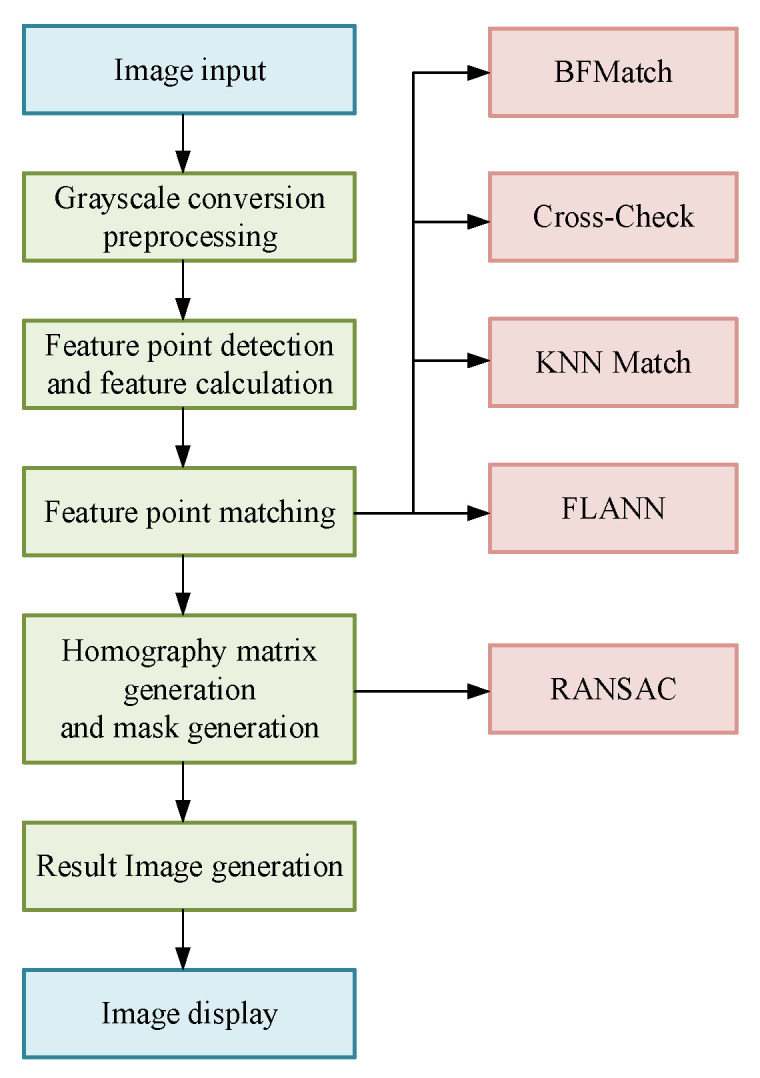
Functional architecture of the system.

To enable automated soldering and image-guided alignment of large PCBs, this paper develops a robot-based PCB image registration system, as illustrated in Figure 1. The system first captures an overall PCB reference image and local sensed images of target soldering regions. Feature matching is then used to estimate the homography between the reference and sensed images. Finally, the registration result is transferred to the robotic arm to support precise welding. As shown in Figure 2, the software workflow includes image acquisition and preprocessing, SIFT feature extraction, feature matching, homography estimation with mask generation, result-image generation and visual display. A ray-casting-based accuracy assessment module is also implemented to quantify the correctness of matched points.

### 2.1. Hardware Design and Implementation

To ensure practical applicability, a dedicated hardware platform was developed for stable image acquisition and robotic operation. The platform integrates a six-axis robotic arm, a customized end effector, a laser displacement sensor, an imaging camera module, a ring-shaped illumination module and an edge-computing/host-PC processing unit into a unified workflow.

**Figure 3:**
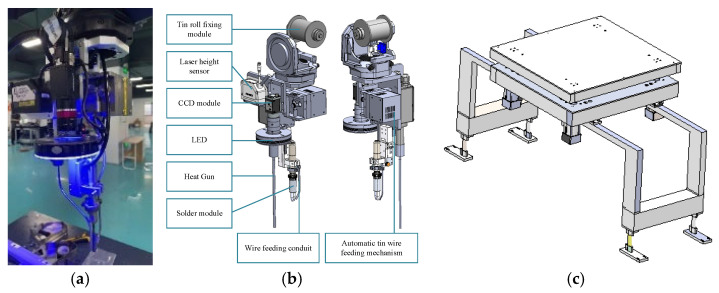
Large-scale PCB robotic welding system: (**a**) physical drawing of the end effector; (**b**) schematic diagram of the end effector structure; (**c**) schematic diagram of the lifting mechanism.

The six-axis robotic arm serves as the core actuator. The customized end effector integrates the laser displacement sensor, imaging camera module and soldering unit (Figure 3), enabling height measurement, image acquisition and welding within the same robotic framework. A ring-shaped blue LED array is mounted around the camera lens to provide directional and uniform illumination. This configuration reduces shadows on uneven PCB surfaces, improves contrast on green PCB substrates and suppresses specular reflection, thereby facilitating more stable SIFT feature extraction.

In addition, a lifting mechanism was incorporated into the double-speed conveyor line to temporarily decouple the fixture board from conveyor vibration, thereby providing a stable platform for visual positioning and alignment. Preliminary image preprocessing is performed on the edge-computing unit, while the full LAKNN–SIFT registration and experimental evaluation are executed on the host PC when higher computational resources are required.

The image acquisition conditions were specified as follows. A Sony FCB-EW9500H color camera (Sony Corporation, Tokyo, Japan) block equipped with a 1/1.8-type STARVIS CMOS sensor was used for image acquisition. To avoid variations caused by automatic optical adjustment, the zoom, focus, exposure and illumination settings were fixed during each group of experiments. The image size used for registration was 2448 × 2048 pixels after cropping, and the exposure time was fixed at 8 ms. Illumination was provided by a ring-shaped blue LED array mounted around the camera lens. The LED array had a peak wavelength of 465 nm, and the illuminance at the PCB surface was approximately 12,000 lux. The effective spatial resolution was estimated according to the calibrated field coverage at each acquisition height. The estimated resolutions corresponding to acquisition heights of 1, 2, 3, 5, 8, 11 and 14 cm were 0.0062, 0.0124, 0.0186, 0.0310, 0.0496, 0.0682 and 0.0868 mm/pixel, respectively.

Before the registration experiments, a preliminary imaging-quality study was performed to validate the acquisition setup. Candidate lighting and acquisition-height settings were compared using the number of detected SIFT key points, the variance of the Laplacian for image sharpness, and the saturated/specular pixel ratio. Under the selected setup, the SIFT detector produced an average of 1847 ± 126 key points per image on the current test image set. The variance of the Laplacian reached 312.6 ± 28.4, indicating sufficient image sharpness, and the saturated/specular pixel ratio was 1.8% ± 0.4%, where saturated pixels were defined as pixels with gray values greater than or equal to 250. These results support the selected acquisition height and lighting configuration.

### 2.2. Image Acquisition and Processing

In the proposed registration workflow, the reference image is either a design drawing or a standard PCB template, whereas the sensed image is captured during production. Because the present study focuses on structural features rather than color information, the images are converted to grayscale before SIFT feature extraction. This conversion reduces color-channel interference and highlights edges, corners and local intensity changes that are important for image alignment.

(i) Image acquisition: The reference and sensed images are loaded using the image-processing library in BGR format and then converted to grayscale. In the revised experiment description, the camera model, sensor, lighting configuration, acquisition height, image size and spatial resolution are explicitly reported to ensure reproducibility.

(ii) Grayscale preprocessing: Grayscale images contain luminance information only, which reduces computational complexity and makes feature extraction less sensitive to color variations caused by illumination. This step improves the stability of subsequent SIFT descriptor extraction and matching.

### 2.3. Feature-Point Detection and Descriptor Extraction

#### 2.3.1. Construction of Scale Space

The SIFT algorithm constructs a scale space by applying Gaussian blurring at multiple levels to generate an image pyramid [22]. Each layer of the pyramid corresponds to a different degree of Gaussian smoothing applied to the original image. The specific scale space is constructed as follows:

(1) $$L\left(x,y,\sigma \right)=G\left(x,y,\sigma \right)*I\left(x,y\right)$$

Here, $L\left(x,y,\sigma \right)$ is the scale-space representation of the image. $G\left(x,y,\sigma \right)$ is a Gaussian kernel function. $I\left(x,y\right)$ is the original image, * denotes the convolution operation, and σ is the standard deviation of the Gaussian blur that controls the degree of blurring.

#### 2.3.2. Key Point Detection

In the constructed scale space, potential key points are identified by searching for extreme points (i.e., local maxima or minima within the scale-space neighborhood) [23]. Each key point corresponds to a local extremum in scale space, typically associated with regions of high contrast or significant intensity variations. Specifically, each point is compared to its 8 neighbors on the same scale, as well as the 9 corresponding pixels in the scales above and below (3 × 3 neighborhoods in adjacent scales). If the point is a maximum or minimum among all 26 neighbors, it is considered a potential key point. If this point is a local maximum or minimum across all these neighborhoods, it is identified as a potential key point.

#### 2.3.3. Precise Location of Key Points

For each detected extremum, the SIFT algorithm refines its location by fitting a 3D quadratic function to interpolate the position and scale, achieving sub-pixel and sub-scale accuracy [24]. This process serves to suppress potential false positives and ensure precise localization of the true key point within the image.

#### 2.3.4. Direction Assignment

To achieve rotation invariance, each feature point is assigned a dominant orientation based on the gradient directions in its local neighborhood. Specifically, for each feature point, the histogram of the gradient direction is computed in its neighborhood, and the largest peak in the histogram is selected as the main direction of the point.

The formula for calculating the gradient is as follows:

(2) $$Gradient\left(x,y\right)=\left(\frac{\partial I}{\partial x},\frac{\partial I}{\partial y}\right)$$

Here, $\frac{\partial I}{\partial x}$ and $\frac{\partial I}{\partial y}$ are the gradients of the image in the x and y directions, respectively. The gradient direction is:

(3) $$\theta =\mathrm{atan}2\left(\frac{\partial I}{\partial y},\frac{\partial I}{\partial x}\right)$$

This step ensures that the feature descriptors are invariant to image rotation.

#### 2.3.5. Feature Descriptor Computation

To facilitate image matching, SIFT generates a descriptor for each key point that captures the gradient distribution in its local neighborhood. Typically, the SIFT descriptor is extracted from a fixed 16 × 16 pixel region centered on the key point. This region is subdivided into 16 smaller blocks, and within each block, an 8-bin histogram of gradient directions is computed. Concatenating these histograms produces a 128-dimensional feature vector that robustly represents the local image structure.

The computation process for each descriptor can be represented as follows:

(4) $$D=\left[{Hist}_{1},{Hist}_{2},\ldots ,{Hist}_{16}\right]$$

Here, ${Hist}_{i}$ is the gradient orientation histogram of the ith sub-region, containing 8 bins representing accumulated gradient magnitudes in each direction. Eventually, the gradient information of the 16 sub-regions is stitched into a 128-dimensional vector.

### 2.4. Feature-Point Matching

Feature-point matching is a crucial step in image registration. In this module, several common matching methods are implemented, including BFMatcher [25], FLANN [26] and KNN [27] (see Section 3). Their effectiveness is evaluated experimentally using the same PCB image pairs and the same inlier-match-ratio metric.

### 2.5. Homography Matrix Generation and Mask Generation

Based on the matched points, the homography matrix—i.e., the projective transformation from the reference image to the sensed image—is estimated using the RANSAC algorithm (see Section 3). RANSAC iteratively samples subsets of the matched point pairs, estimates candidate homography matrices and identifies inliers that are consistent with the projective model [28].

Each inlier correspondence imposes the following homogeneous constraint on the homography matrix:

(5) $$x_{obj}\times \left(Hx_{ref}\right)=0$$

Here, ${{x_{ref}=[x}_{ref}{,y}_{ref},1]}^{T}$ and ${{x_{obj}=[x}_{obj}{,y}_{obj},1]}^{T}$ denote the homogeneous coordinate vectors of the corresponding points in the reference and sensed images, respectively. H is the 3×3 homography matrix, and × denotes the vector cross product. Thus, Equation (5) expresses the collinearity constraint used to estimate the homography matrix from the inlier correspondences.

Mask generation is employed to spatially mark inliers (valid matching points) identified by the RANSAC algorithm. Specifically, a binary mask image is constructed, with pixels corresponding to inliers set to 1 (foreground) and the others to 0 (background). This mask serves two key roles: (i) it filters out outliers during the homography matrix application, ensuring only reliable feature points contribute to the image transformation; (ii) it facilitates visualization of matching quality by highlighting valid correspondences, aiding in qualitative evaluation of registration results. The mask is generated synchronously with homography matrix estimation using the inlier indices from RANSAC to map valid points to the image coordinate system.

### 2.6. Resulting Image Generation

The estimated homography matrix defines the forward mapping from the reference-image coordinate system to the sensed-image coordinate system. For a point in the reference image, the corresponding point in the sensed image is obtained by applying the homography matrix and normalizing the resulting homogeneous coordinates:

(6) $$x_{obj}=\frac{Hx_{ref}}{{\left(Hx_{ref}\right)}_{3}}$$

Equation (6) obtains the Cartesian image coordinates by normalizing the transformed homogeneous vector with its third component. To generate the final registered image, the sensed image is warped into the reference-image coordinate system using the inverse homography matrix. The transformed image can then be superimposed on the reference image using an image-fusion method, such as weighted averaging. The inverse mapping is expressed as follows:

(7) $$x_{ref}=H^{-1}\cdot x_{obj}$$

### 2.7. Accuracy Assessment Based on the Ray Method

To evaluate alignment accuracy, the ray-casting method [29] is employed to determine whether matched points fall within a specified region of the sensed image. The ray-casting method determines whether a point falls within a region by shooting a ray from the point to be tested and checking whether it intersects with the boundary of the target region. The distance from the point to the boundary of the target region is calculated and compared with a set threshold. If the distance from the point to the region boundary is below the threshold, the point is considered a correct match. The matching accuracy is then computed as the ratio of correct matches to total matches:

(8) $$Accuracy=\frac{\mathrm{correct}\;\mathrm{matches}}{\mathrm{total}\;\mathrm{matches}}\times 100\%$$

where “correct matches” refers to the number of point pairs that meet the distance threshold and “total matches” is the total number of matched point pairs.

## 3. Proposed Image Registration Method

### 3.1. Introduction to Traditional Image Registration Methods

#### 3.1.1. Brute-Force Matching (BF Match)

The brute-force matching method is a fundamental method for matching 2D feature points. For each descriptor $R_{i}$ in the first image, brute-force matching computes its distance to all descriptors ${\{S}_{1},S_{2},{\ldots ,S}_{n},\}$ in the second image and then selects the most similar descriptor $S_{j}$ as the candidate match. The Euclidean distance is used to measure similarity between descriptors:

(9) $$d_{\mathrm{Euclidean}}(R_{i},S_{j})=\sqrt{\sum_{k=1}^{d}{\left(R_{i}\left(k\right)-S_{j}\left(k\right)\right)}^{2}\;}$$

Here, d is the dimension of the descriptor, and $R_{i}(k)$ and $S_{j}(k)$ denote the value of the kth element in the descriptors $R_{i}$ and $S_{j}$, respectively. The accuracy of matching can be improved to some extent by methods such as cross-validation or setting distance thresholds.

#### 3.1.2. Cross-Matching (Cross-Check)

Cross-check is a strategy used to improve feature-matching accuracy, aiming to filter out incorrect pairs of matches, especially reducing the number of “one-to-many” or “many-to-one” wrong matches. The basic idea is to perform reverse verification after brute-force matching is completed. Specifically, if the descriptor $R_{i}$ in the reference image and the descriptor $S_{j}$in the sensed image are the best match for each other (i.e., $R_{i}$ is the best match for $S_{j}$, and vice versa), they are considered as correct matches. Through reverse verification, cross-validation can effectively exclude matches that occur only in one-way matching, thereby reducing false matches, especially when duplicate features or background interference are present in the image.

#### 3.1.3. Fast Library for Approximate Nearest Neighbors (FLANN)

FLANN is a library designed for fast approximate nearest neighbor searches in large-scale datasets. By constructing efficient data structures, FLANN can greatly accelerate the matching process [30]. Compared to brute-force matching, FLANN demonstrates superior performance on large datasets and is especially well-suited for real-time applications. It commonly employs k-d tree indexing to enable efficient nearest neighbor searches, where the data space is recursively partitioned along different dimensions to speed up the search process [31]. Given a dataset $D=\left\{x_{1},x_{2},\ldots ,x_{n}\right\}$ and a query point $q\in R^{k}$, the k-d tree is constructed as follows:

(i) Selection of dividing dimensions: one dimension $d\in \left\{1,2,\ldots ,k\right\}$ is selected for each layer, step by step, in a cyclic manner;

(ii) Node construction: sort the data points along the selected dimension, and choose the median $x_{m}$ as the node, which partitions the space into left and right subtrees;

(iii) Recursive splitting: repeat the above for the left and right subtrees until the stopping condition is satisfied. The query process aims to find the nearest neighbor $x_{NN}$ of the query point q, minimizing the distance function:

(10) $$d\left(q,x_{NN}\right)=\underset{x_{i}\in D}{min}∥q-x_{i}∥)$$

Here, $∥q-x_{i}∥$ is the Euclidean distance as follows:

(11) $$∥q-x_{i}∥=\sqrt{\sum_{d=1}^{k}{\left(q_{d}-x_{id}\right)}^{2}}$$

By traversing the k-d tree, FLANN can achieve a fast approximate nearest neighbor search.

#### 3.1.4. Random Sample Consensus (RANSAC)

RANSAC is a classical parameter estimation method for robust model fitting in the presence of outliers. The basic idea is to iteratively sample random subsets of the data to estimate a model and then identify inliers—points consistent with the model—by evaluating the model against the entire dataset.

In feature matching, RANSAC can effectively remove the wrong matching points. The process includes calculating the single response matrix between two images based on the matching points and further decomposing the rotation matrix R and translation vector t of the camera [32]. The 3D coordinates of the matching points are estimated via triangulation. These 3D points are then projected back onto the image plane, and the reprojection error is computed. If the error between the projected coordinates and the original observation is below a predefined threshold, the matched point pair is classified as an inlier.

#### 3.1.5. K-Nearest Neighbors Matching (KNN Match)

The K-nearest neighbor matching algorithm is used to improve the matching accuracy by selecting the first k-nearest neighbors of the feature points and performing distance-ratio filtering. For each reference feature point $R_{i}$, the algorithm first identifies the two nearest neighbors in the sensed image: $S_{j1}$ (the closest match) and $S_{j2}$ (the second closest match). The pair ($R_{i},S_{j1}$) is accepted as a valid match only if the distance ratio between the optimal and suboptimal matches is below a predefined threshold $t\in \left[0.5,0.9\right]$. This ratio test helps ensure matching distinctiveness.

The distance ratio is computed for each reference feature point $R_{i}$ and its two nearest neighbors, $S_{j1}$ (the nearest neighbor) and $S_{j2}$ (the second-nearest neighbor), in the target image:

(12) $$r_{i}=\frac{d\left(R_{i},S_{j1}\right)}{d\left(R_{i},S_{j2}\right)}$$

where $d\left(R_{i},S_{j1}\right)$ and $d\left(R_{i},S_{j2}\right)$ denote the Euclidean distances from $R_{i}$ to its nearest and second-nearest neighbors, respectively. The binary matching-decision indicator $A_{i}$ is defined as

(13) $$A_{i}=\left\{\begin{array}{ll} 1, & if{\;r}_{i}<t\\ 0, & ifr_{i}\geq t \end{array}\right.$$

where t is the predefined distance-ratio threshold. $A_{i}$ = 1 indicates that the candidate match $\left(R_{i},S_{j1}\right)$ is accepted, whereas $A_{i}$ = 0 indicates that it is rejected. A smaller distance ratio means that the nearest neighbor is substantially more similar to $R_{i}$ than the second-nearest neighbor, thereby reducing ambiguous matches and improving matching reliability.

### 3.2. PCB Image Registration Method Based on LAKNN and SIFT

In traditional KNN-based image registration methods, a fixed K value is commonly used. However, when applied to complex PCB images, this fixed setting may reduce matching accuracy. To address this issue, this paper presents an optimized KNN registration algorithm that adaptively adjusts the K value based on local density estimation and matching confidence. The proposed method proceeds through four main steps: (i) SIFT feature extraction, where feature points and their descriptors are extracted from the images; (ii) LAKNN matching, in which the K value is dynamically adjusted according to local feature density and matching confidence; (iii) mismatch elimination using the RANSAC algorithm; and (iv) affine transformation and final registration output.

#### 3.2.1. Adaptive K Value Collaborative Adjustment Strategy

Specifically, the kernel density estimation method is adopted to estimate the local density of a certain feature point x, and the K value is dynamically adjusted in combination with its matching confidence [33]. The local density estimation is calculated using the Gaussian kernel function as follows:

(14) $$\hat{\rho }\left(x\right)=\frac{1}{n}\sum_{i=1}^{n}\exp\left(-\frac{∥x-x_{i}{∥}^{2}}{2h^{2}}\right)$$

Here, $x_{i}$ is the neighbor of the feature point x, and h is the bandwidth parameter of the kernel function. This density reflects the local density of the current feature points in space.

Matching confidence is used to measure the degree of credibility between a certain feature point and its best matching point and is defined as follows:

(15) $$C_{i}=1-\frac{d_{1}}{d_{2}}$$

Here, $d_{1}$ and $d_{2}$ represent the Euclidean distances between the current point and its best match and suboptimal match. When $C_{i}$ is larger, it indicates a more reliable match.

The joint adjustment strategy for the K value is as follows:

(16) $$k_{local}\left(x\right)=round\left(k_{base}\left(1+\alpha \left(1-\hat{\rho }\left(x\right)\right)\right)\left(1-\beta C_{i}\right)\right)$$

Here, $k_{base}$ is the base K value, and α and β are hyperparameters used to balance the influence of local density and matching confidence.

This strategy achieves a decrease in K when the feature-point density is high and an increase in K when the matching confidence is low, thereby enhancing the adaptability of KNN matching to different image regions. The advantage of the proposed strategy over standard nearest neighbor (NN) matching or fixed ratio tests is particularly evident in complex PCB images. The feature density varies drastically between integrated circuit (IC) pins (high density) and empty substrates (low density). A fixed k value or a static ratio threshold inevitably leads to either missed matches in dense areas or false positives in sparse areas. The proposed LAKNN strategy effectively relaxes constraints in sparse regions and tightens in dense regions, mimicking human visual confirmation mechanisms.

Regarding the hyperparameters in Equation (16), α and β determine the sensitivity of the adaptive k value to the local density ($\rho_{x}$) and matching confidence ($C_{i}$), respectively. These parameters were determined empirically through a grid search experiment on a validation set consisting of 50 representative PCB images. During the optimization process, we varied $\alpha \in \;\left[0.5,\;2.0\right]$ and $\beta \in \;\left[0.1,\;0.9\right]$. The optimal combination (α=1.0, β=0.5) was selected as it maximized the inlier-match ratio while maintaining a recall rate above 85%.

#### 3.2.2. Characteristic Distance Calculation and Matching Discrimination

To evaluate the similarity between SIFT feature points, this paper adopts Euclidean distance as the measurement method of feature descriptors. For two N-dimensional SIFT feature descriptors $f_{1}$ and $f_{2}$ (N=128), the distance is defined as:

(17) $$d\left(f_{1},f_{2}\right)=\sqrt{\sum_{j=1}^{N}{\left(f_{1j}-f_{2j}\right)}^{2}}$$

In KNN matching, we find the top K neighbors with the smallest distances for each feature point and then use the distance-ratio criterion for validity screening. Only when the following conditions are satisfied will the matched pair be retained:

(18) $$\frac{d_{1}}{d_{2}}<\tau$$

Here, the distance-ratio threshold τ was set to 0.75. This setting follows Lowe’s classical SIFT ratio-test principle [12], in which a candidate match is accepted only if the nearest neighbor descriptor distance is sufficiently smaller than the second-nearest neighbor distance. To verify its suitability for PCB images, a threshold-sensitivity experiment was conducted at τ = 0.65, 0.70, 0.75, 0.80 and 0.85. The results showed that τ = 0.75 provided the best overall trade-off between false matches and missed matches under the present PCB imaging conditions, achieving an inlier ratio of 81.3%, a false-match rate of 6.2% and a recall of 89.7%. Although larger thresholds retained more candidate matches, they also introduced more ambiguous correspondences, while smaller thresholds removed many valid matches and reduced recall. Therefore, τ = 0.75 was used in all subsequent experiments.

In summary, the proposed LAKNN–SIFT method not only maintains high precision but also significantly enhances robustness against complex PCB images with rotations, scaling variations, and multi-view scenarios. Its core innovation lies in the LAKNN strategy, which jointly leverages kernel density estimation of feature distributions and confidence-guided thresholding to dynamically adjust the matching process. Unlike conventional fixed-parameter KNN approaches, this adaptive mechanism fully accounts for local differences in feature-point distributions and matching confidence, ensuring stable performance across diverse and challenging PCB registration conditions.

From an industrial perspective, the proposed method is embedded into a hardware--software co-designed platform that includes a robotic arm, adaptive illumination, and edge computing units. This integration enables closed-loop PCB inspection and automated soldering operations, demonstrating the practical feasibility of the approach beyond algorithmic enhancement.

## 4. Experimental Verification

### 4.1. Dataset, Target PCB and Evaluation Setup

**Figure 4:**
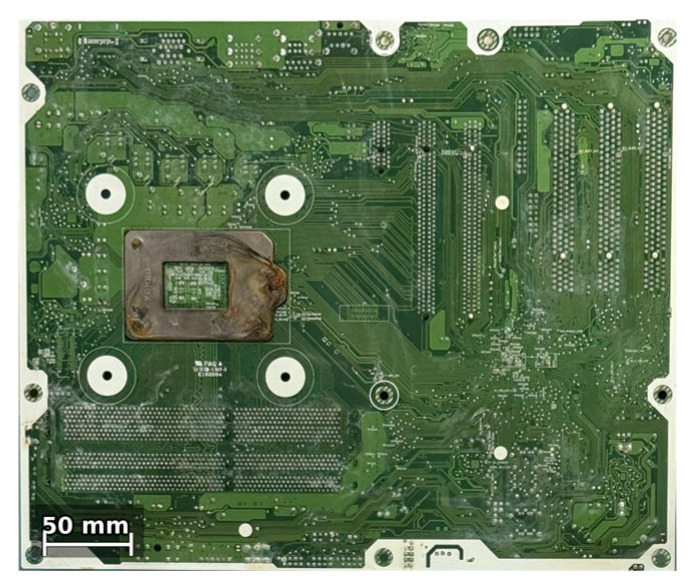
Reference image.

The target specimen was a four-layer FR4 industrial control motherboard with physical dimensions of 380 mm × 280 mm × 1.6 mm. It contained QFP packages, BGA components, 0402 passive elements, through-hole connectors, repeated solder pads, connector-pin arrays, and regions with both dense and sparse feature distributions. This PCB was selected for its large size and highly heterogeneous component density. It includes dense IC pad clusters alongside sparse connector regions. These characteristics provide a representative and rigorous test case for evaluating adaptive feature-matching performance under realistic industrial conditions. The reference image was obtained from a whole-board scan, whereas the sensed images were captured using the camera system under controlled acquisition-height and viewpoint conditions. The four local regions in Figure 4 were selected to cover feature-rich, feature-sparse and nonuniform feature-distribution cases.

In total, 20 unique image pairs were used in the main comparative experiments, comprising 7 pairs for acquisition-height evaluation, 4 pairs for regional robustness evaluation, and 9 viewpoint pairs. The 9 viewpoint pairs were evaluated in both forward and reverse matching directions at angles of 0°, 2°, 3°, 5°, 10°, 15°, 20°, 25° and 30°, resulting in 18 registration evaluations. Thus, the complete experimental suite includes 29 registration evaluations in total. For the representative PCB images, scale bars were added according to the image type and the calibrated spatial resolution. Board-level scale bars were used for whole-board, registration-effect and region-selection images, while local-scale bars were used for local sensed images. The spatial resolution was reported according to the corresponding acquisition height. This experimental design enabled a systematic evaluation of the proposed LAKNN method under changes in image scale, local feature density and viewpoint deflection.

All computations were performed on an Intel Core i7-12700H CPU @ 2.30 GHz with 32 GB DDR5 memory. No dedicated GPU acceleration was used for the registration pipeline. The experiments were conducted under Ubuntu 20.04 LTS using Python 3.9 and OpenCV 4.8.0. Runtime was measured as the average over five independent runs for BF, BF_CrossCheck, FLANN, KNN_Low, KNN_High and LAKNN under identical hardware and software conditions.

### 4.2. Experiment 1: PCB Images for Different Heights

In this experiment, the reference target described in Section 4.1 was used, as shown in Figure 4. The reference image was obtained by scanning the whole PCB at 600 dpi, and the image size used for registration was 2448 × 2048 pixels after cropping. The sensed images were captured using a Sony FCB-EW9500H color camera block (Sony Corporation, Tokyo, Japan) equipped with a 1/1.8-type STARVIS CMOS sensor. To ensure consistent acquisition conditions, the zoom, focus, exposure and illumination settings were fixed during each group of experiments. The spatial resolutions corresponding to the tested acquisition heights are reported in Section 2.1.

**Figure 5:**
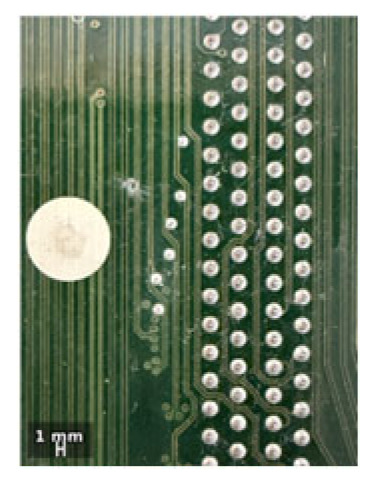
Sensed image with a height of 5 cm.

**Figure 6:**
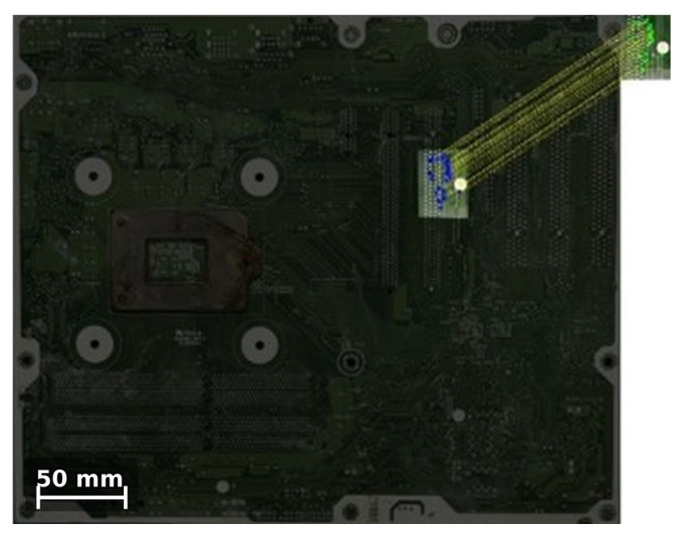
PCB registration effect diagram (with a height of 5 cm as an example).

Figure 5 shows a representative sensed image captured at an acquisition height of 5 cm. Figure 6 illustrates the corresponding registration result, in which the sensed image is transformed into the reference-image coordinate system. The close overlap of the board contours and component structures provides a qualitative indication that the estimated homography yields effective alignment under this representative acquisition condition.

In practical applications, the camera installation height affects image scale, feature size and registration accuracy [34]. Therefore, sensed images were captured at *Z*-axis acquisition heights of 1 cm, 2 cm, 3 cm, 5 cm, 8 cm, 11 cm and 14 cm. Scale bars were added to the representative PCB images according to the spatial scale of each figure, and the inlier-match ratio was used as the primary evaluation metric.

**Figure 7:**
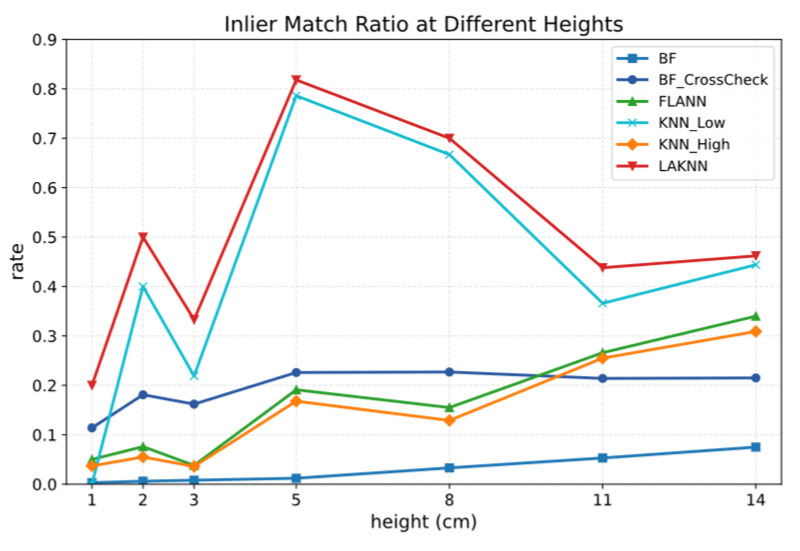
Inlier-match accuracy point-line diagram at different heights.

From Figure 7 and Table 1, the proposed LAKNN method achieves the highest inlier ratio across all tested heights with a clear peak around 5–8 cm (81.8% and 70.0%). Performance degrades at extreme close-range and long-range acquisitions—expected due to scale variation and loss of detail—but LAKNN still maintains better robustness than all baseline methods across the tested range. The arithmetic mean across the seven discrete heights is 49.3%.

### 4.3. Experiment 2: PCB Images for Different Regions

**Figure 8:**
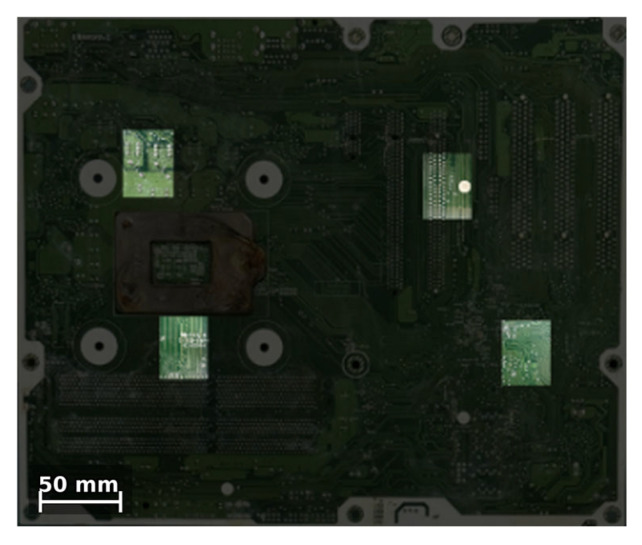
PCB different area selection diagram.

**Figure 9:**
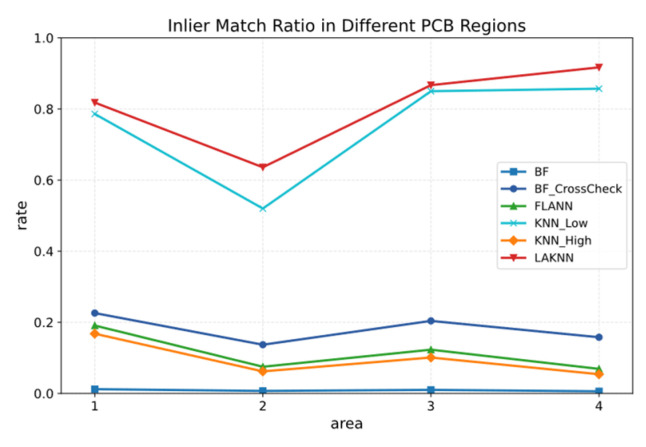
Inlier-match accuracy point-line graph in different areas.

To verify the robustness of the proposed LAKNN method, four different regions of the reference image were also selected for this study, as shown in Figure 8. Six image registration methods were used for comparison in the experiment. The results are shown in Figure 9 and Table 2. It is noteworthy that KNN_Low achieves competitive results in Areas 3 and 4 (85.0% and 85.7%). This is likely because these regions contain highly distinctive, locally clustered features that align well with a small, fixed neighborhood size. However, its performance drops drastically in Area 2 (52.0%) and under viewpoint changes (Table 3), confirming that a fixed K value lacks the robustness required for heterogeneous PCBs. This observation further validates the necessity of the proposed adaptive strategy.

### 4.4. Experiment 3: PCB Images from Different Perspectives

To address potential camera viewing-angle deflection in practical applications, this study also investigates the accuracy of various methods across different viewing angles [35]. In practical application, when aligning the components or the details of the solder joints, the *Z*-axis acquisition height is usually about 5–8 cm, so 5 cm was selected as the standard height for testing viewing-angle variations, which were also evaluated across six experimental groups.

In image registration, perspective deflection frequently hinders feature-point matching, especially under large-angle deflection, where the mismatch rate increases. To systematically evaluate robustness against viewpoint changes, both forward and reverse matching were performed. In reverse matching, the reference image and sensed image are exchanged, which provides a control experiment for evaluating descriptor consistency and registration stability under viewpoint deflection. Additional measurements at 25° and 30° were included to verify whether the accuracy trend continues beyond 20°.

**Figure 10:**
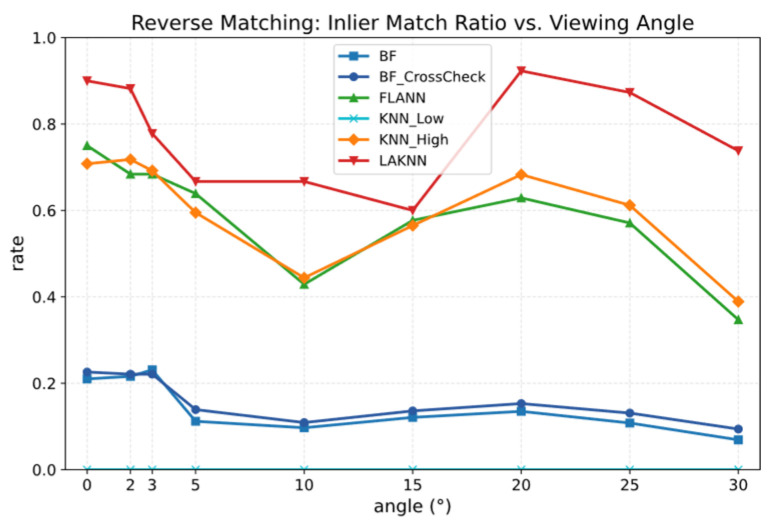
Inlier-match accuracy point-line graph from different perspectives (reverse).

The results in Figure 10 and Table 3 show that the accuracy of traditional matching methods, such as BF and KNN_High, gradually decreases as the viewing angle increases from 0° to 30°. At a 20° reverse-viewing-angle deflection, the BF and KNN_High accuracies are 13.5% and 68.3%, respectively, while the proposed LAKNN method reaches 92.3%. The additional measurements at 25° and 30° further show that LAKNN maintains higher accuracy than the baseline methods, with inlier-match ratios of 87.3% and 73.8%, respectively. The KNN_Low method failed to produce stable matches in the reverse matching experiments, as indicated by N/A in Table 3. These results demonstrate that LAKNN provides more reliable registration under large viewpoint deflections.

**Figure 11:**
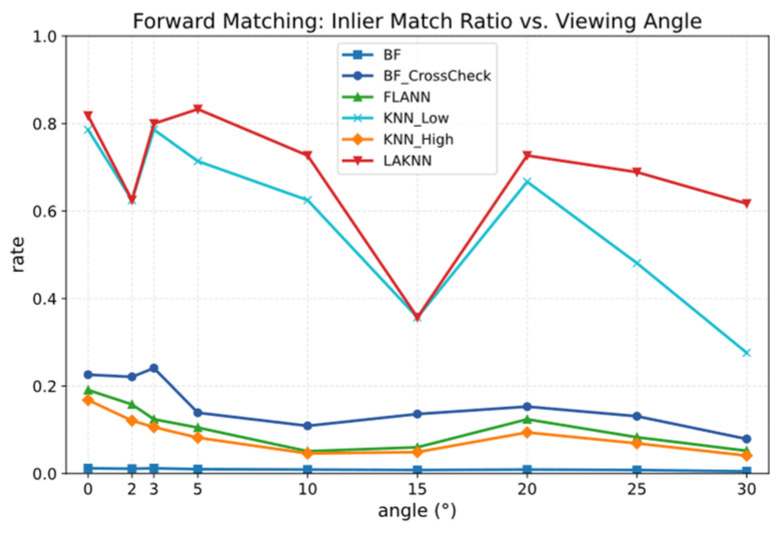
Inlier-match accuracy point-line graph from different perspectives (positive).

In the forward matching experiments, Figure 11 and Table 4 show that LAKNN performs well under small viewing-angle deflections from 0° to 5°, with an inlier-match accuracy of 81.8% at 0° and 83.3% at 5°. As the viewing angle increases, the forward matching accuracy decreases; at 20°, it remains 72.7%, and at 25° and 30° conditions, it reaches 68.9% and 61.7%, respectively. Although the forward accuracy is lower than the reverse-matching result at large angles, it remains higher than or comparable to the other traditional methods.

Overall, forward matching with LAKNN performs well under small-angle deflections (0°–5°), while reverse matching shows stronger robustness under larger angles (≥15°). This suggests that two-way verification could further improve registration robustness in practice.

### 4.5. Ablation Experiment

To validate the effectiveness of the adaptive K-value strategy and matching confidence adjustment mechanism in the proposed LAKNN algorithm, this section conducts an ablation study. The LAKNN-full method is compared with its simplified variants to evaluate the individual contributions of key modules to the overall performance.

Four methods’ variants were designed for comparison: (1) LAKNN-Full. The LAKNN-full method incorporates both the adaptive K-value adjustment and matching confidence weighting strategies. (2) LAKNN-FixedK. A variant with the adaptive K-value strategy removed (K is fixed at 2), but the matching confidence mechanism is retained. (3) LAKNN-NoConf. Variant with the matching confidence strategy removed while retaining the adaptive K-value adjustment. (4) KNN-Basic. The conventional KNN-based matching method has a fixed K-value and no matching confidence mechanism.

All variants were evaluated on 20 registration trials captured at a height of 5 cm under standard viewing conditions. The inlier-match ratio was used as the primary accuracy metric, and average matching time was measured over five independent trials to reflect computational efficiency. The same host-PC configuration described in Section 4.1 was used for all variants.

**Figure 12:**
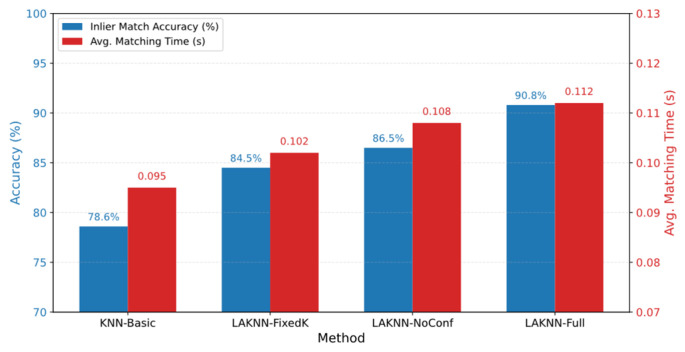
Performance comparison of LAKNN variants in the ablation study. The average matching time includes natural fluctuations caused by variations in local feature distributions and hardware processing load. Each result is averaged over five independent trials.

The ablation results in Table 5 and Figure 12 show that both the adaptive K-value strategy and the matching-confidence mechanism improve registration performance. Removing either module leads to a decline in accuracy, confirming their individual contributions. Compared with KNN-Basic, LAKNN-NoConf improves accuracy by 7.9 percentage points, LAKNN-FixedK improves accuracy by 5.9 percentage points, and the full LAKNN method improves accuracy by 12.2 percentage points, reaching 90.8%. Although the full model requires slightly more matching time, the accuracy gain is important for PCB inspection tasks where incorrect localization may lead to welding errors.

To assess the computational overhead of the proposed method against the six matching baselines, Table 6 compares their mean matching times under identical hardware conditions. LAKNN requires 0.112 s per match, which is 39.8% faster than BF and only 0.016 s slower than KNN_Low. Although FLANN is the fastest method (0.082 s), the accuracy results in Section 4.2, Section 4.3 and Section 4.4 show that its lower runtime is accompanied by lower inlier-match ratios. Thus, LAKNN provides a practical balance between registration accuracy and computational cost.

### 4.6. Discussion of Hardware–Software Synergy

The superior registration performance reported in this study is attributed to the synergy between stable image acquisition and algorithmic adaptation. The lifting mechanism reduces platform vibration and motion blur, while the ring-shaped blue LED illumination reduces shadows and specular reflection. These hardware improvements provide higher-quality input images for SIFT feature extraction. However, hardware improvements alone do not resolve geometric distortion and repeated local patterns. As shown in Table 5, KNN-Basic reaches 78.6% accuracy under the same acquisition conditions, whereas the full LAKNN method reaches 90.8%. Therefore, the hardware ensures data stability, and the LAKNN algorithm improves final registration precision. To quantify the hardware contribution, blur and reflection were measured using the variance of the Laplacian and the saturated-pixel ratio, respectively. Compared with a single fixed white-light source without the ring-shaped blue LED array, the proposed setup changed these metrics from 184.3 ± 21.7 and 7.6% ± 1.1% to 312.6 ± 28.4 and 1.8% ± 0.4%, respectively. This indicates improved sharpness and a lower proportion of saturated/specular pixels under the proposed acquisition setup.

## 5. Conclusions and Future Work

This paper proposes a hardware–software integrated PCB image registration method based on LAKNN and SIFT. By combining stable robotic image acquisition with locally adaptive feature matching, the method improves registration robustness under height variation, regional feature-density variation and viewing-angle deflection. The experimental results show that LAKNN achieves higher inlier-match ratios than BF, FLANN and fixed-parameter KNN baselines under the tested conditions.

Future work will focus on three directions. First, the hardware platform will be deployed on real PCB assembly lines for long-term validation under production conditions. Second, a larger and more diverse PCB dataset will be constructed, covering different board types, component layouts, lighting conditions and manufacturers. Third, controlled experiments under different blur, reflection and production-vibration levels will be further expanded to evaluate robustness and computational cost in real industrial environments.

## Figures and Tables

**Table 1:** Inlier-match accuracy record at different heights.

Rate	BF	BF_CrossCheck	FLANN	KNN_Low	KNN_High	LAKNN
1 cm	0.3%	11.4%	5.0%	0.0%	3.7%	**20** **.** **0** **%**
2 cm	0.6%	18.1%	7.6%	40.0%	5.5%	**50** **.** **0** **%**
3 cm	0.8%	16.2%	3.8%	21.9%	3.6%	**33** **.** **3** **%**
5 cm	1.2%	22.6%	19.1%	78.6%	16.8%	**81** **.** **8** **%**
8 cm	3.3%	22.7%	15.5%	66.7%	12.9%	**70** **.** **0** **%**
11 cm	5.3%	21.4%	26.6%	36.6%	25.5%	**43** **.** **8** **%**
14 cm	7.5%	21.5%	34.0%	44.4%	30.9%	**46** **.** **2** **%**
Average	2.7%	19.1%	15.9%	41.2%	14.1%	**49** **.** **3** **%**

**Table 2:** Inlier-match accuracy record in different areas.

Rate	BF	BF_CrossCheck	FLANN	KNN_Low	KNN_High	LAKNN
Area1	1.2%	22.6%	19.1%	78.6%	16.8%	**81** **.** **8** **%**
Area2	0.7%	13.7%	7.5%	52.0%	6.2%	**63** **.** **6** **%**
Area3	1.0%	20.4%	12.3%	85.0%	10.1%	**86** **.** **7** **%**
Area4	0.6%	15.8%	6.9%	85.7%	5.4%	**91** **.** **7** **%**
Average	0.9%	18.1%	11.5%	75.3%	9.6%	**81** **.** **0** **%**

**Table 3:** Inlier-match accuracy record from different perspectives (reverse).

Rate	BF	BF_CrossCheck	FLANN	KNN_Low	KNN_High	LAKNN
0°	21.0%	22.6%	75.0%	N/A	70.8%	**90** **.** **0** **%**
2°	21.6%	22.1%	68.4%	N/A	71.8%	**88** **.** **2** **%**
3°	23.1%	22.1%	68.4%	N/A	69.2%	**77** **.** **8** **%**
5°	11.2%	13.9%	63.9%	N/A	59.5%	**66** **.** **7** **%**
10°	9.7%	10.9%	42.9%	N/A	44.4%	**66** **.** **7** **%**
15°	12.1%	13.6%	57.7%	N/A	56.5%	**60** **.** **0** **%**
20°	13.5%	15.3%	62.9%	N/A	68.3%	**92** **.** **3** **%**
25°	10.8%	13.1%	57.1%	N/A	61.2%	**87** **.** **3** **%**
30°	6.9%	9.4%	34.7%	N/A	38.9%	**73** **.** **8** **%**
Average	14.4%	15.9%	59.0%	N/A	60.1%	**78** **.** **1** **%**

**Table 4:** Inlier-match accuracy record from different perspectives (positive).

Rate	BF	BF_CrossCheck	FLANN	KNN_Low	KNN_High	LAKNN
0°	1.2%	22.6%	19.1%	78.6%	16.8%	**81.8**%
2°	1.1%	22.1%	15.8%	62.5%	12.1%	**62.5**%
3°	1.2%	24.1%	12.4%	78.6%	10.6%	**80.0**%
5°	1.0%	13.9%	10.5%	71.4%	8.2%	**83.3**%
10°	0.9%	10.9%	5.1%	62.5%	4.6%	**72.7**%
15°	0.8%	13.6%	6.0%	35.7%	4.9%	**35.7**%
20°	0.9%	15.3%	12.4%	66.7%	9.4%	**72.7**%
25°	0.8%	13.1%	8.3%	48.1%	6.9%	**68.9**%
30°	0.5%	7.9%	5.2%	27.6%	4.1%	**61.7**%
Average	0.9%	15.9%	10.5%	59.1%	8.6%	**68.8**%

**Table 5:** Ablation results and approximate matching times of LAKNN variants.

Method	Adaptive K	Confidence Weighting	Inlier Accuracy	Avg. Matching Time	Improvement
KNN-Basic	No	No	78.6%	0.095 s	Baseline
LAKNN-FixedK	No	Yes	84.5%	0.102 s	+5.9 p.p.
LAKNN-NoConf	Yes	No	86.5%	0.108 s	+7.9 p.p.
LAKNN-Full	Yes	Yes	90.8%	0.112 s	+12.2 p.p.

**Table 6:** Computational cost comparison under identical hardware conditions.

Method	Mean Matching Time	Relative Change vs. BF
BF	0.186 s	Baseline
BF_CrossCheck	0.228 s	+22.6%
FLANN	0.082 s	−55.9%
KNN_Low	0.096 s	−48.4%
KNN_High	0.101 s	−45.7%
LAKNN	0.112 s	−39.8%

## Data Availability

The original contributions presented in this study are included in the article. Further inquiries can be directed to the corresponding author.
